# Supplementary material for: Neurofibromin 1 in mushroom body neurons mediates circadian wake drive through activating cAMP–PKA signaling
Source: Nat Commun. 2021 Oct 1;12:5758. doi: 10.1038/s41467-021-26031-2 (PMC8486785; doi:10.1038/s41467-021-26031-2)
Supplement: Supplementary file 1 — Supplementary information [file 41467_2021_26031_MOESM1_ESM.pdf]

SUPPLEMENTARY INFORMATION

Supplementary Figures

Supplementary Figure 1

Supplementary Figure 2

Supplementary Figure 3

Supplementary Figure 4

Supplementary Figure 5

Supplementary Table titles and legends

Supplementary Table 1

Supplementary Table 2

Supplementary Table 3

Supplementary Table 4

Supplementary Table 5

Supplementary Table 6

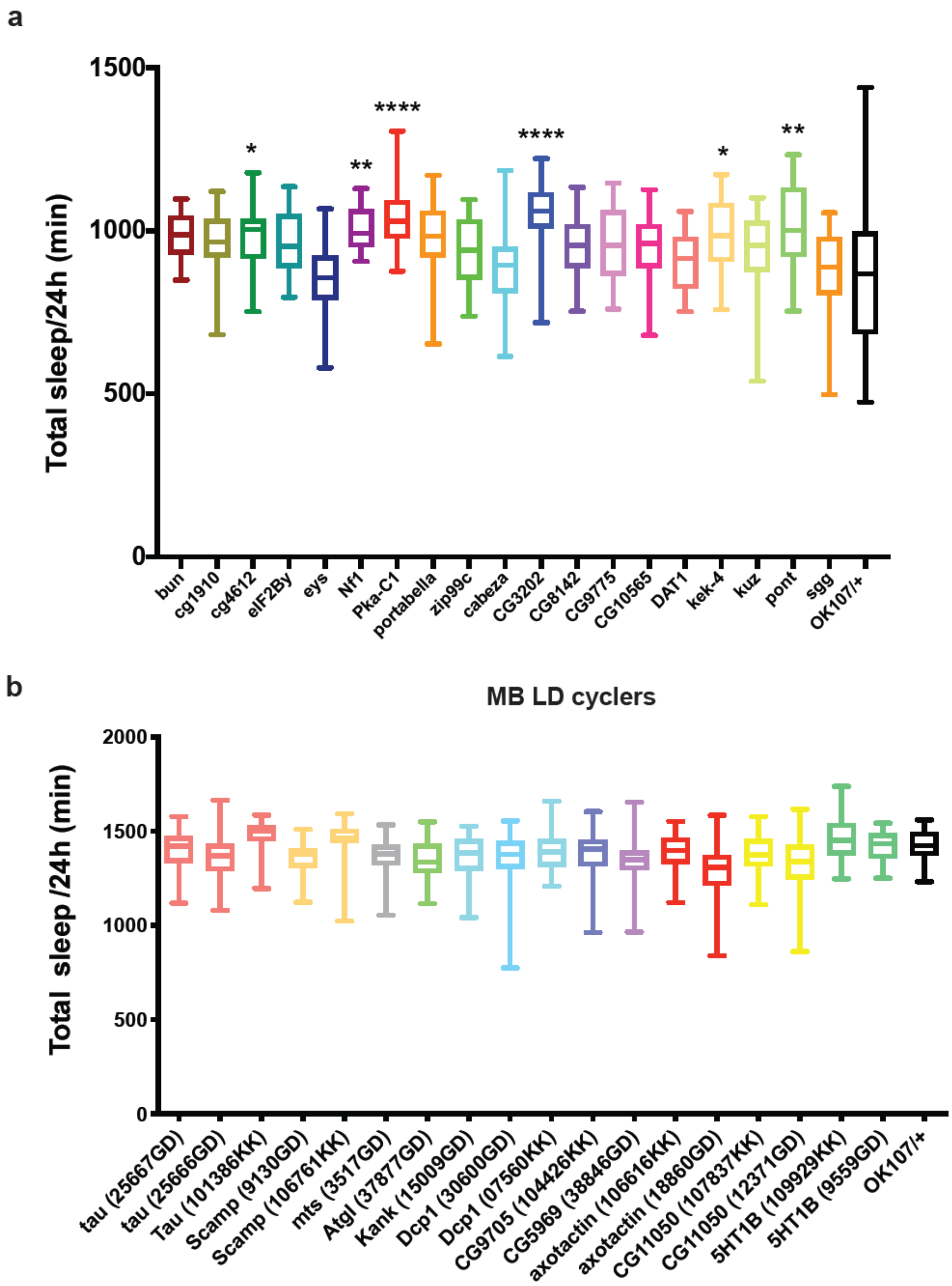

Supplementary Figure 1. RNAi screening of the MB LD cyclers for sleep
a. Boxplot of the daily total sleep of female flies expressing an RNAi transgene with the OK107 driver. The center lines indicate the median, box boundaries are 25th and 27th percentiles, and the whiskers extend to the minimum and maximum values. The total amount of sleep per day was compared with *OK107/+* by The Kruskal-Wallis one-way ANOVA with Dunnett's multiple comparisons test (**p* < 0.05, ***p* < 0.01, *****p* < 0.0001; n = 21–46 flies per group). **b.** Results of the screening of the MB LD cyclers for sleep. The boxplot represents the total sleep amount as in **a.** Each gene was knocked down with the OK107 driver. No significant change in total sleep amount was detected by ANOVA with Dunnett's multiple comparisons test. Source data are provided as a Source Data file.

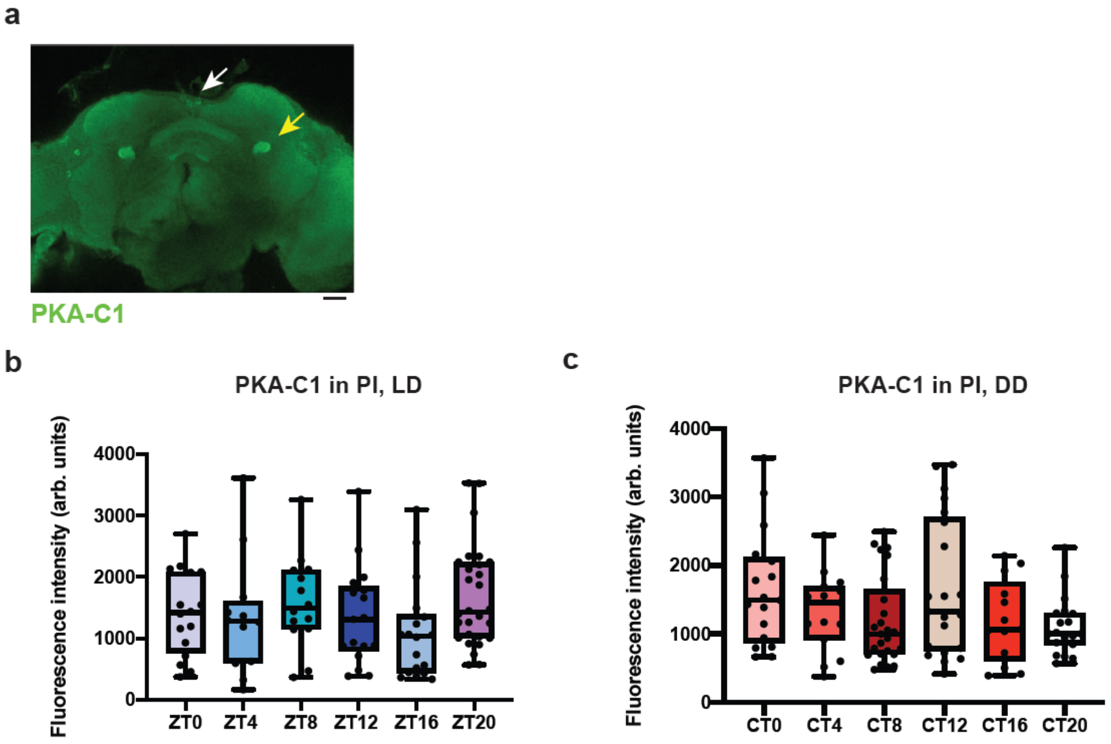

Supplementary Figure 2. Pka-C1 expression in the PI does not oscillate
a. A representative confocal image of anti-Pka-C1 staining in the brain acquired at CT8. White arrow indicates the Pka-C1 signal in the PI. Yellow arrow indicates the calyx of the MB. Note that the image is a posterior view of the brain and thus Pka-C1 expression in the MB lobes is not visible. Scale bar, 25 μm. **b and c.** Quantification of the Pka-C1 levels in the PI during LD (**b**) and DD (**c**). The center lines indicate the median, box boundaries are the 25th and 75th percentiles, and the whiskers represent the minimum and maximum values. arb. units, arbitrary units. n=20-30 per group. Source data are provided as a Source Data file.

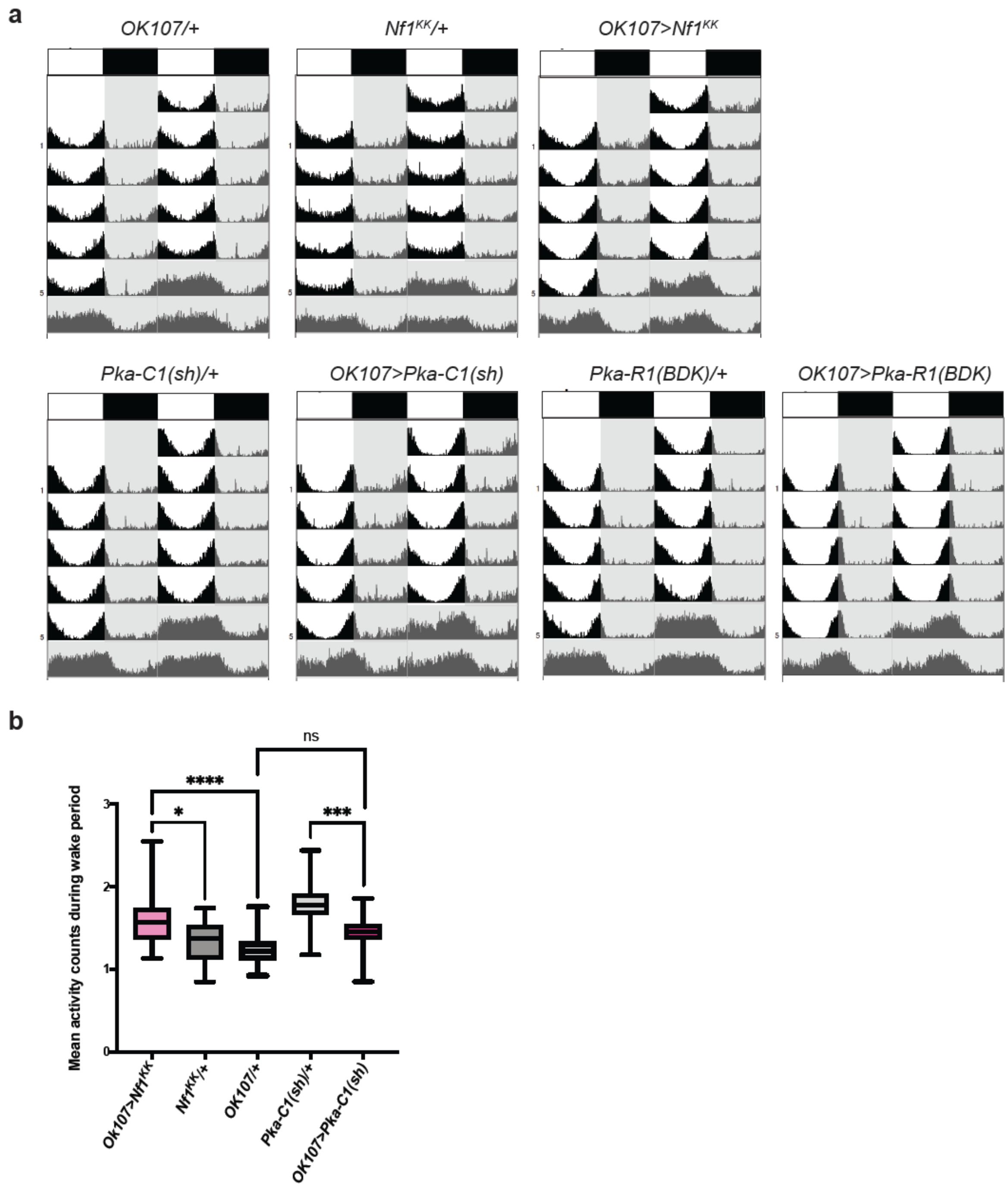

Supplementary Figure 3. Inhibition of *Nf1* or *Pka-C1* in the MB does not affect locomotor activity

a. Double-plotted actograms of flies of indicated genotypes. Group average activities of virgin female flies (n= 28-32 per genotype) during 5 days in DD and 2 days in DD are shown. White and black boxes indicate light (white) and dark (black) period in 12h:12h-LD cycles, and gray shades indicate the period of darkness. **b.** Mean activity counts during wake period in LD in the virgin female flies of indicated genotypes. The center lines of the box plots indicate the median, box boundaries are 25th and 27th percentiles, and the whiskers represent the minimum and maximum values. n=28-32 per group. **p* < 0.05, ***p < 0.001, ****p < 0.0001 by the Kruskal-Wallis one-way ANOVA with Dunn's multiple comparisons test. Source data are provided as a Source Data file.

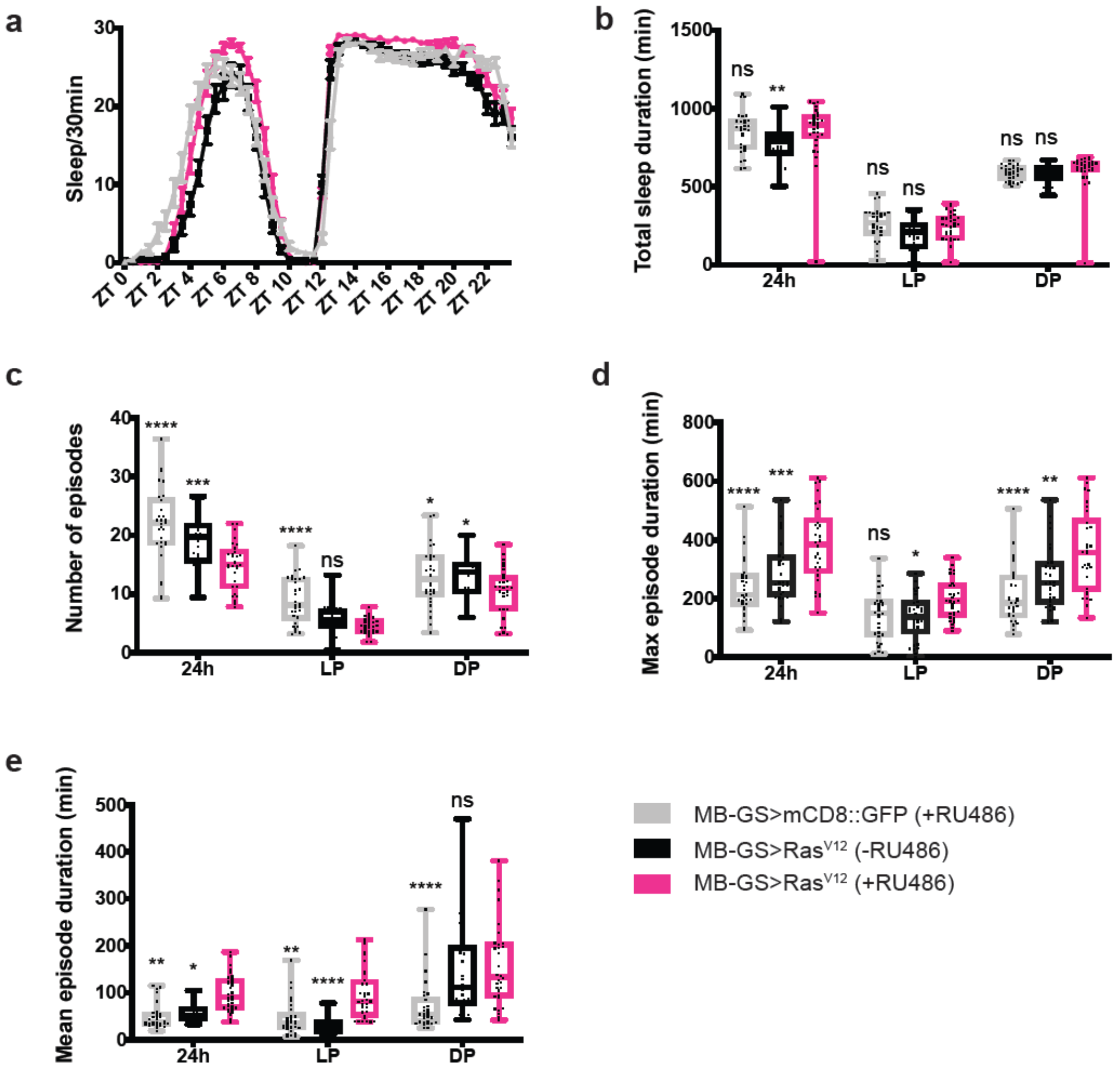

Supplementary Figure 4. Ras hyperactivation in the adult MB

Sleep profiles, amount, and structure of *MB-GS > Ras*^{v12} and *MB-GS > mCD8:GFP* flies fed with RU486 (+RU486) or those without RU486 (-RU486) during adulthood (n = 30 per group). *MB-GS > Ras*^{v12}(+RU486) was compared with other groups by the two-way ANOVA with Dunnett's multiple comparisons test. $*p < 0.05$, $**p < 0.01$, $***p < 0.001$, and $****p < 0.0001$). The center lines of the box plots indicate the median, box boundaries are 25th and 27th percentiles, and the whiskers represent the minimum and maximum values. **a.** Sleep per 30-min bin averaged over 5 days of LD. **b.** Total sleep duration. **c.** Number of sleep episodes. Data are mean ± SEM. **d.** Maximum sleep episode duration. **e.** Mean sleep episode duration. Source data are provided as a Source Data file.

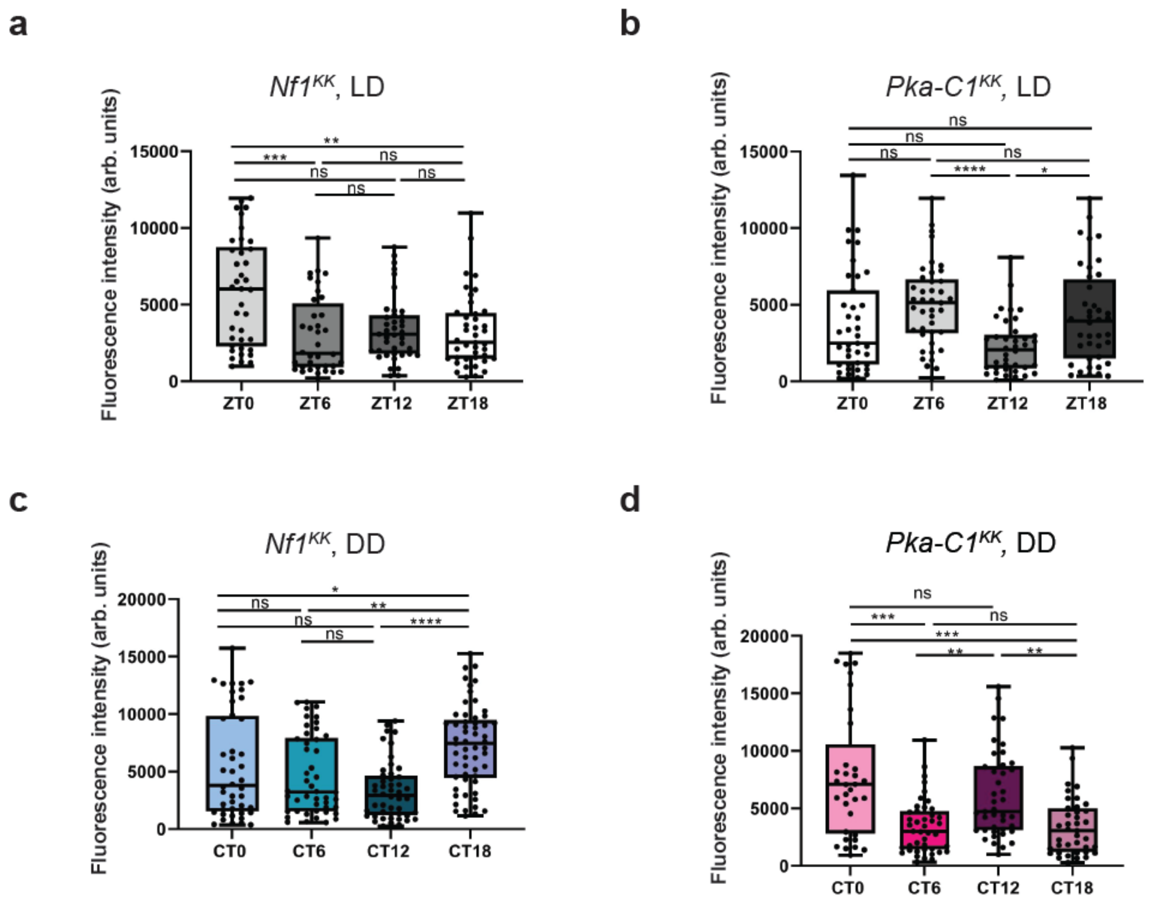

Supplementary Figure 5. NF1 and PKA-C1 mediate calcium rhythms in the MB
Relative calcium levels in the MB lobes are monitored by expressing CaLexA-GFP with *30Y-GAL4* together with *UAS-Nf1*^{KK} or *UAS-Pka-C1*^{KK}. Quantification of GFP intensity across all MB lobes in LD with *Nf1* RNAi (**a**), in LD with *Pka-C1* RNAi (**b**), in DD with *Nf1* RNAi (**c**) and in DD with *Pka-C1* RNAi (**d**). n= 40-50. The center lines of the box plots indicate the median, box boundaries represent the 25th and 75th percentiles, and the whiskers represent the minimum and maximum values. Stars indicate statistical significance levels comparing values between timepoints using the non-parametric Kruskal-Wallis one-way ANOVA with Dunn's multiple comparison's test (*$p < 0.05$, **$p < 0.01$, ***$p < 0.001$ and ****$p < 0.0001$). arb. units, arbitrary units. Source data are provided as a Source Data file.

Supplementary Table 1. Gene Ontology analysis of the MB LD-DD cyclers
A total of 117 MB LD-DD cyclers were subjected to the GO term enrichment analysis using the GOrilla tool.

GO term	Description	P-value	FDR q-value	Genes	
GO:0050890	cognition	2.37E-04	1.00E+00	*CG1910*	*prt*
				w	*Pka-C1*
				Nf1	*CG4612*
				sgg	
GO:0007611	learning or memory	2.37E-04	8.92E-01	*CG1910*	*prt*
				w	*Pka-C1*
				Nf1	*CG4612*
				sgg	
GO:0016070	RNA metabolic process	2.60E-04	6.51E-01	*TfIIFbeta*	*Ars2*
				caz	*CG4935*
				YT521-B	*CG5800*
				CG2263	*Mat1*
				CG16912 (α-PheRS)	*CG11417*
				CG2972	*CG4973*(*mdlc*)
				CG14450	*wuho*
				Taf8	*Clp*
				Ref1	
GO:0008355	olfactory learning	2.89E-04	5.44E-01	*prt*	*Pka-C1*
				Nf1	*sgg*
GO:0008306	associative learning	6.59E-04	9.92E-01	*prt*	*Pka-C1*
				Nf1	*sgg*
GO:0015842	aminergic neurotransmitter loading into synaptic vesicle	7.02E-04	8.80E-01	*prt*	*w*
GO:0015837	amine transport	7.02E-04	7.55E-01	*prt*	*w*
GO:0006367	transcription initiation from RNA polymerase II promoter	8.35E-04	7.85E-01	*TfIIFbeta*	*caz*
				Mat1	*Taf8*

Supplementary Table 2. Enriched transcription factor binding sites in the promoters of the MB LD-DD cyclers

Top 30 transcription factor (TF) binding sites over-represented in the promoter regions of the MB LD-DD cycling genes, sorted by the Z-score.

TF	JASPAR ID	Class	Family	Z-score	Fisher score
**Optix**	MA0199.1	Helix-Turn-Helix	Homeo domain	**7.245**	**1.414**
**twi**	MA0249.1	Zipper-type	Helix-Loop-Helix	**6.817**	**0.646**
**onecut**	MA0235.1	Helix-Turn-Helix	Homeo	**6.159**	**0.577**
hb	MA0049.1	Zinc-coordinating	β-β-α-zinc finger	**5.922**	0.132
**dl_1**	MA0022.1	Ig-fold	Rel	**5.91**	**1.493**
br_Z1	MA0010.1	Zinc-coordinating	β-β-α-zinc fingerfinger	**5.853**	0.217
fkh	MA0446.1	Winged Helix-Turn-Helix	Forkhead	**5.667**	0.349
z	MA0255.1	Helix-Turn-Helix	Zeste	**5.516**	0.081
so	MA0246.1	Helix-Turn-Helix	Homeo domain	4.759	**0.599**
brk	MA0213.1	Helix-Turn-Helix	Brinker	4.534	**1.119**
Six4	MA0204.1	Helix-Turn-Helix	Homeo domain	3.302	0.328
bap	MA0211.1	Helix-Turn-Helix	Homeo domain	3.242	**0.769**
br_Z3	MA0012.1	Zinc-coordinating	β-β-α-zinc fingerfinger	2.916	0.359
Lag1	MA0193.1	Helix-Turn-Helix	Homeo domain	2.849	0.057
hkb	MA0450.1	Zinc-coordinating	β-β-α-zinc fingerf	2.452	0.196
CG42234	MA0174.1	Helix-Turn-Helix	Homeo domain	2.382	0.271
usp	MA0016.1	Zinc-coordinating	Hormone-nuclear Receptor	2.001	**0.565**
br_Z4	MA0013.1	Zinc-coordinating	β-β-α-zinc finger	1.97	0.016
Cf2_II	MA0015.1	Zinc-coordinating	β-β-α-zinc finger	1.846	0.104
D	MA0445.1	Other Alpha-Helix	High Mobility Group	1.775	0.088
Su(H)	MA0085.1	Other	LAG1	1.705	**0.663**
CG34031	MA0444.1	Helix-Turn-Helix	Homeo domain	1.704	0.046
vvl	MA0254.1	Helix-Turn-Helix	Homeo domain	1.623	**0.854**
slp1	MA0458.1	Winged Helix-Turn-Helix	Forkhead	1.571	0.133
Hmx	MA0192.1	Helix-Turn-Helix	Homeo domain	1.483	0.063
Kr	MA0452.1	Zinc-coordinating	β-β-α-zinc finger	1.426	**0.715**
ct	MA0218.1	Helix-Turn-Helix	Homeo domain	1.407	0.018
Gsc	MA0190.1	Helix-Turn-Helix	Homeo domain	1.357	0.171
achi	MA0207.1	Helix-Turn-Helix	Homeo domain	1.297	0.176
ovo	MA0126.1	Zinc-coordinating	β-β-α-zinc finger	1.291	0.063

Significantly enriched TF binding sites that fulfil both a Z-score > 5.5 and a Fisher score > 0.5 are marked in bold.

Supplementary Table 3. Predicted targets of circadian-relevant miRNAs among the MB LD-DD cyclers

miRNA	Target					
Bantam	*sgg*	*kuz*	*sky*	*Clamp*	*Nf1*	*Cnx99A*	
Let-7	*sgg*	*kek4*					
miR-279	*CG33523*	*sky*	*Act88F*	*ste14*	*Cnx99A*		
miR-276a	*anne*	*Nf1*					
miR-210	*Pka-C1*	*rhomboid-4*	*cactus*				
miR-263b	*CG10508*	*Ythdc1*	*w*	*Nf1*	*qm*	*Ref1*	
miR-124	*pck*	*CG15877*	*CG10916*	*CG3409*			
miR-964	*nkd*	*CG1910*	*CG2972*	*CG5180*	*CG4953*	*CG5498*	*CG10916*
miR-263a	*sgg*	*CG4975*	*sky*	*pck*	*ZIPIC*	*Clamp*	*CG2972*
	Act88F	*wde*	*TfIIFβ*	*Pka-C1*	*eIF2Bγ*	*CG3409*	*Pat1*
	Ref1						

miRNAs known to be expressed rhythmically in the fly brain are marked in bold.

Supplementary Table 4. List of the MB LD-DD cyclers selected for RNAi sleep screening
LD adj.p and DD adj.p indicate adjusted *p*-values reported by the non-parametric JTK_Cycle in LD and DD, respectively. Gene snapshot describes the key information on the known function and biological roles of the gene.

Flybase ID	Gene symbol	LD adj.p	DD adj.p	Gene snapshot
FBgn0000273	*Pka−C1*	1.34E-02	8.83E-03	Protein kinase, cAMP-dependent, catalytic subunit 1. Axis specification, rhythmic behavior, synaptic transmission.
FBgn0003371	*sgg*	8.83E-03	4.30E-02	Glycogen synthase kinase 3. A component of the β-catenin destruction complex in the canonical Wnt pathway. Circadian rhythms.
FBgn0015269	*Nf1*	4.30E-02	4.85E-04	GTPase activating protein. Development, postembryonic growth, learning and memory, circadian rhythms.
FBgn0022349	*CG1910*	1.38E-03	1.34E-02	Long-term memory.
FBgn0031414	*eys*	2.01E-02	8.83E-03	Temperature entrainment of circadian rhythms.
FBgn0035016	*CG4612*	8.83E-03	5.71E-03	Long-term memory, perception of pain.
FBgn0039714	*Zip99C*	3.62E-03	3.62E-03	Zinc/iron regulated transporter-related protein 99C.
FBgn0043005	*prt*	3.62E-03	4.85E-04	Vesicular transporter. Expressed in the mushroom body.
FBgn0259176	*bun*	8.83E-03	4.30E-02	Mushroom body development, sleep.
FBgn0034029	*eIF2Bγ*	5.12E-07	3.62E-03	Eukaryotic translation initiation factor 2B subunit gamma
FBgn0032484	*kek4*	4.30E-02	1.55E-04	Transmembrane leucine-rich repeat (LRR) and immunoglobulin-like domain-containing (LIG) protein
FBgn0033309	*CG8735*	8.83E-03	8.83E-03	Unknown
FBgn0037261	*CG9775*	3.62E-03	8.83E-03	Unknown
FBgn0259984	*kuz*	2.01E-02	8.83E-03	ADAM metalloendopeptidase. Axon guidance.
FBgn0037538	*CG3223*	2.01E-02	4.30E-02	Unknown
FBgn0037051	*CG10565*	1.55E-04	1.55E-04	Unknown
FBgn0029878	*Pat1*	2.01E-02	1.38E-03	Regulation of microtubule-based movement. Localizes to the kinesin complex.
FBgn0040078	*pont*	2.01E-02	1.38E-03	AAA+ (ATPases Associated with various cellular Activities) family.
FBgn0030007	*α-PheRS*	4.30E-02	1.55E-04	Phenylalanyl-tRNA synthetase, α-subunit.
FBgn0035016	*CG4612*	8.83E-03	5.71E-03	Predicted 3'UTR binding. Long-term memory.
FBgn0030871	*CG8142*	8.83E-03	8.83E-03	Unknown

Supplementary Table 5. Circadian locomotor rhythmicity of the flies following the knockdown of the MB LD-DD cyclers in the MB

Some genotypes displayed reduced rhythmicity (**p* < 0.05, ***p* < 0.01, ****p* < 0.001 according to the chi-square test, compared with *OK107-GAL4/+*). No significant difference in the period (Tau ± SEM) was seen by knockdown as compared with *OK107-GAL4/+* flies (one-way ANOVA with Dunnett's multiple comparison test).

Genotype		% Rhythmic	Tau ± SEM (h)	Power ± SEM
OK107>UAS-RNAi	*Pka-C1*	96.8	23.8 ± 0.05	161.2 ± 8.45
	Nf1	87.5	23.8 ± 0.04	153.8 ± 7.28
	CG1910	71.9*	23.8 ± 0.05	116.1 ± 8.13
	eys	93.8	24.0 ± 0.02	161.6 ± 9.13
	zip99c	87.5	23.8 ± 0.05	149 ± 11.4
	portabella	100	23.9 ± 0.05	133.8 ± 8.11
	bun	90.6	24.2 ± 0.41	105.7 ± 7.13
	CG4616	81.5	24.3 ± 0.54	127.8 ± 10.18
	eIF2betgam	90.6	23.9 ± 0.03	165.9 ± 9.52
	hk2	87.5	23.9 ± 0.08	167.9 ± 18.25
	CG9775	73.7*	23.2 ± 0.21	42.8 ± 4.62
	kuz	73.9*	23.4 ± 0.09	36 ± 1.77
	CG3202	32.1***	23.4 ± 0.11	36.7 ± 5.98
	CG10565	57.7**	23.2 ± 0.11	38.3 ± 3.45
	Dat1	70.6*	23.4 ± 0.06	49 ± 4.19
	pont	36.4***	23.4 ± 0.13	40.6 ± 3.78
	alpha-PheRs	93.3	23.4 ± 0.12	42.4 ± 5.22
	CG8142	50***	23.3 ± 0.1	34.9 ± 2.77
	cabeza	44.8***	23.6 ± 0.1	33.6 ± 2.82
	kek-4	61.9***	23.5 ± 0.17	36.9 ± 3.54
Controls	w^{1118}	96.9	23.4 ± 0.05	51.2 ± 3.43
	OK107/+	92.6	23.6 ± 0.05	662.3 ± 56.93

Supplementary Table 6. Fly strains used in this study

Strains from the Bloomington Drosophila Stock Center	Stock number
201Y	BDSC_4440
c747	BDSC_6494
c309	BDSC_6906
c739	BDSC_7362
1471	BDSC_9465
30Y	BDSC_30818
MB247	BDSC_50742
H24	BDSC_51632
17D	BDSC_51631
OK107	BDSC_854
MB-Gene-Switch	BDSC_81013
UAS-Epac1-cAMPs	BDSC_25409
w^{*}*; P{LexAop-CD8-GFP-2A-CD8-GFP}2; P{UAS-mLexA-VP16-NFAT}H2, P{lexAop-rCD2-GFP}3/TM6B, Tb*^{1}	BDSC_66542
**Strains from the Vienna Drosophila Resource Center**	**Stock number**
UAS-Pka−C1 RNAi	101524 KK
UAS-Nf1 RNAi	109637 KK
UAS-CG1910 RNAi	40159 GD
UAS-Eys RNAi	106892 KK
UAS-CG4612 RNAi	52497 GD
UAS-Zip99C RNAi	1362 GD
UAS-Portabella RNAi	104763 KK
UAS-Bun RNAi	19679 GD
UAS-eIF2Bγ RNAi	43917 GD
UAS-kek4 RNAI	105647 KK
UAS-CG9775 RNAI	108437 KK
UAS-kuzbanian RNAI	107036 KK
UAS-CG3223 RNAi	104063 KK
UAS-CG10565 RNAi	105149 KK
UAS-Pat1 RNAi	27307 GD
UAS-pont RNAi	105408 KK
UAS-α-PheRS RNAi	33514 GD
UAS-CG4612 RNAi	52497 GD
UAS-CG8142 RNAi	108452 KK
UAS-cabeza RNAi	100291 KK
UAS-Pka-C1 (*shRNA*)	330111
UAS-Tau RNAi	25667 GD
UAS-Tau RNAi	25666 GD
UAS-Tau RNAi Tau(II)_(101386KK)	101386 KK
UAS-Scamp RNAi	9130 GD
UAS-Scamp RNAi	06761 KK
UAS-Mts-RNAi	35171 GD
UAS-Kank RNAi	15009 GD
UAS-Hdac RNAi	30600 GD
UAS-Dcp1 RNAi	107560 KK
UAS-CG9705 RNAi	104426KK
UAS-CG5968 RNAi	38846 GD
UAS-CG11050 RNAi	38846GD
UAS-Axotactin RNAi	1066616 KK
UAS-ATGL RNAi	37877 GD

UAS-5HT1B RNAi	109929 KK
UAS-5HT1B RNAi	9559 GD
Other	Reference
UAS-mcD8::GFP	Lee, T. and Luo, L. 1999
