# Supplementary material for: Neurofibromin 1 in mushroom body neurons mediates circadian wake drive through activating cAMP–PKA signaling
Source: Nat Commun. 2021 Oct 1;12:5758. doi: 10.1038/s41467-021-26031-2 (PMC8486785; doi:10.1038/s41467-021-26031-2)
Supplement: Supplementary file 2 — Reporting Summary [file 41467_2021_26031_MOESM2_ESM.pdf]

nature research

Corresponding author(s): Emi Nagoshi

Last updated by author(s): Sep 9, 2021

Reporting Summary

Nature Research wishes to improve the reproducibility of the work that we publish. This form provides structure for consistency and transparency in reporting. For further information on Nature Research policies, see Authors & Referees and the Editorial Policy Checklist .

Statistics

For all statistical analyses, confirm that the following items are present in the figure legend, table legend, main text, or Methods section.

n/a	Confirmed	
[ ]	[x]	The exact sample size (*n*) for each experimental group/condition, given as a discrete number and unit of measurement
[ ]	[x]	A statement on whether measurements were taken from distinct samples or whether the same sample was measured repeatedly
[ ]	[x]	The statistical test(s) used AND whether they are one- or two-sided<br>*Only common tests should be described solely by name; describe more complex techniques in the Methods section.*
[ ]	[x]	A description of all covariates tested
[ ]	[x]	A description of any assumptions or corrections, such as tests of normality and adjustment for multiple comparisons
[ ]	[x]	A full description of the statistical parameters including central tendency (e.g. means) or other basic estimates (e.g. regression coefficient) AND variation (e.g. standard deviation) or associated estimates of uncertainty (e.g. confidence intervals)
[ ]	[x]	For null hypothesis testing, the test statistic (e.g. *F*, *t*, *r*) with confidence intervals, effect sizes, degrees of freedom and *P* value noted<br>*Give P values as exact values whenever suitable.*
[x]	[ ]	For Bayesian analysis, information on the choice of priors and Markov chain Monte Carlo settings
[x]	[ ]	For hierarchical and complex designs, identification of the appropriate level for tests and full reporting of outcomes
[x]	[ ]	Estimates of effect sizes (e.g. Cohen's *d*, Pearson's *r*), indicating how they were calculated

Our web collection on statistics for biologists contains articles on many of the points above.

Software and code

Policy information about availability of computer code

Data collection	DAMsystem 3 (Trikinetics)
Data analysis	JTK_CYCLE (v3.1), GOrilla, oPOSSUM (v3.0), microRNA.org, SCAMP (v2), Image J/Fiji (v2.1.0/1.53c), STAR (v2.7.9.a), featureCounts function of the Rsubread package (v1.22.2), ggplot2 in R (v3.1.0), GraphPad Prism (v.8.1), MATLAB (MathWorks, version R2017a, build 9.2.0.538062)

For manuscripts utilizing custom algorithms or software that are central to the research but not yet described in published literature, software must be made available to editors/reviewers. We strongly encourage code deposition in a community repository (e.g. GitHub). See the Nature Research guidelines for submitting code & software for further information.

Data

Policy information about availability of data

All manuscripts must include a data availability statement. This statement should provide the following information, where applicable:

- Accession codes, unique identifiers, or web links for publicly available datasets
- A list of figures that have associated raw data
- A description of any restrictions on data availability

Source data are provided with this paper. RNA-seq data are deposited in the Sequence Read Archive (SRA) (https://www.ncbi.nlm.nih.gov/sra) as a Bioproject PRJNA719003 (https://www.ncbi.nlm.nih.gov/bioproject/?term=PRJNA719003),
with the accession numbers SRR14127352- SRR14127363, SRR14127675- SRR14127686, and SRR14213543- SRR14213554. Detailed information of the RNA-seq samples and their corresponding accession numbers are included in the Source Data. The databases used in this study are microRNA.org (http://www.microrna.org/), oPOSSUM (v3.0) (http://opossum.cisreg.ca/oPOSSUM3/), and Gorilla (http://cbl-gorilla.cs.technion.ac.il/).

Field-specific reporting

Please select the one below that is the best fit for your research. If you are not sure, read the appropriate sections before making your selection.

- [x] Life sciences
- [ ] Behavioural & social sciences
- [ ] Ecological, evolutionary & environmental sciences

For a reference copy of the document with all sections, see nature.com/documents/nr-reporting-summary-flat.pdf

Life sciences study design

All studies must disclose on these points even when the disclosure is negative.

Sample size	Sample sizes were chosen without calculation but based on the literature describing similar experiments. Sample sizes for sleep experiments are similar to those in (Parisky et al. Curr Bio 2016, Guo et al. Nature 2016). Sample sizes for the immunostaining, calcium imaging and cAMP imaging experiments were ≥ 20 brains per group, which exceed the range of the sample sizes used for similar experiments in the literature, such as (Chouhan et al. Nature 2020, Bai et al. Cell Reports 2018).
Data exclusions	Data were excluded from the analyses only when they were obtained in the experiments failed technical problems, such as poor RNA-seq quality (Fig. 1b) and low viability of flies (Fig. 2a, Supplementary Fig. 1a, b).
Replication	All the experiment except for the fly sleep experiments presented in Figure 2a and Supplementary Fig. 1a, b, were repeated at least twice. The genetic screens shown in the Figure 2a and Supplementary Fig. 1a and b were not repeated because of the exploratory nature of these experiments. Replicate experiments were successful.
Randomization	Flies were allocated into experimental groups by genotype.
Blinding	Investigators were not blinded to fly genotypes in most experiments, because only one experimenter has conducted the entire course of the experiment, starting from genotyping. However, all data were analyzed using unbiased statistical methods.

Reporting for specific materials, systems and methods

We require information from authors about some types of materials, experimental systems and methods used in many studies. Here, indicate whether each material, system or method listed is relevant to your study. If you are not sure if a list item applies to your research, read the appropriate section before selecting a response.

Materials & experimental systems

n/a	Involved in the study	
[ ]	[x]	Antibodies
[x]	[ ]	Eukaryotic cell lines
[x]	[ ]	Palaeontology
[ ]	[x]	Animals and other organisms
[x]	[ ]	Human research participants
[x]	[ ]	Clinical data

Methods

n/a	Involved in the study	
[x]	[ ]	ChIP-seq
[x]	[ ]	Flow cytometry
[x]	[ ]	MRI-based neuroimaging

Antibodies

Antibodies used	Rabbit Monoclonal anti-GFP (Thermo Fisher, Cat# G10362), mouse anti-Fasciclin II (DSHB, #1D4), Alexa 488 goat anti-rabbit IgG (Thermo Fisher, Cat# A11008), Alexa 633 goat anti-mouse IgG (Thermo Fisher, Cat#A21052), guinea pig anti-PKA-C1 antibodies (gift of Dr. Pascal Therond)
Validation	The antibodies were purchased from Thermo Fisher and DSHB with the exception of anti-PKA-C1, which was a gift from Dr. Pascal Thérond. The antibodies from commercial sources and have been validated by the vendors. Validation data are available on the manufacturer's website: Rabbit Monoclonal anti-GFP (Thermo Fisher, Cat# G10362, https://www.thermofisher.com/antibody/product/GFP-Antibody-Recombinant-Monoclonal/G10362), mouse anti-Fasciclin II (DSHB, #1D4, it was used and validated in 63 different publications as shown in the website: https://dshb.biology.uiowa.edu/1D4-anti-Fascicli-II). Alexa 488 goat anti-rabbit IgG (Thermo Fisher, Cat#A11008, https://www.thermofisher.com/antibody/product/Goat-anti-Rabbit-IgG-H-L-Cross-Adsorbed-Secondary-Antibody-Polyclonal/A-11008), Alexa 633 goat anti-Mouse IgG (Thermo Fisher, Cat#A21052, https://www.thermofisher.com/antibody/product/Goat-anti-Mouse-IgG-H-L-Highly-Cross-Adsorbed-Secondary-Antibody-Polyclonal/A-21052). Guinea pig anti-PKA-C1 was generated and validated in the laboratory of Dr. Pascal Thérond (Ranieri, N., Thérond, P. & Ruel, L. Nat Commun (2014)).

Animals and other organisms

Policy information about studies involving animals; ARRIVE guidelines recommended for reporting animal research

Laboratory animals	Drosophila melanogaster. Male or virgin female flies of 2 to 5-day-old male flies were used in this study. Details of the genotypes, age, and sex are described in the Methods section and in the relevant figure legends.
Wild animals	This study did not use wild animals.
Field-collected samples	The study did not involve samples collected from the field.
Ethics oversight	This study does not involve vertebrate or higher invertebrate animals and does not require ethics oversight.

Note that full information on the approval of the study protocol must also be provided in the manuscript.
