# Supplementary material for: Neurofibromin 1 in mushroom body neurons mediates circadian wake drive through activating cAMP–PKA signaling
Source: Nat Commun. 2021 Oct 1;12:5758. doi: 10.1038/s41467-021-26031-2 (PMC8486785; doi:10.1038/s41467-021-26031-2)
Supplement: Supplementary file 3 — Description of Additional Supplementary Files [file 41467_2021_26031_MOESM3_ESM.pdf]

Title: Supplemental Data 1

Description: Raw read counts and RPKM of RNA-seq data of MB neurons isolated during LD and DD, and the results of cycling expression analysis using JTK_CYCLE.

Title: Supplemental Data 2

Description: Raw read counts and RPKM of RNA-seq data of MB neurons isolated from *per*^{0} mutant flies in DD, and the results of cycling expression analysis using JTK_CYCLE.
